# Prognostic impact and the relevance of PTEN copy number alterations in patients with advanced colorectal cancer (CRC) receiving bevacizumab*

**DOI:** 10.1002/cam4.75

**Published:** 2013-03-25

**Authors:** Timothy J Price, Jennifer E Hardingham, Chee K Lee, Amanda R Townsend, Joseph W Wrin, Kate Wilson, Andrew Weickhardt, Robert J Simes, Carmel Murone, Niall C Tebbutt

**Affiliations:** 1 Haematology-Oncology Department, The Queen Elizabeth HospitalWoodville, SA, 5011, Australia; 2 School of Medicine, University of AdelaideAdelaide, SA, 5000, Australia; 3 School of Medical Sciences, University of AdelaideAdelaide, SA, 5000, Australia; 4 NHMRC Clinical Trials Centre, The University of SydneyCamperdown, NSW, 2050, Australia; 5 Austin HealthHeidelberg, Vic., 3084, Australia; 6 Ludwig Institute for Cancer ResearchHeidelberg, Vic., 3084, Australia

**Keywords:** Bevacizumab, colorectal, KRAS, prognosis, PTEN, VEGF

## Abstract

Loss of phosphatase and tensin homologue (PTEN) expression may be prognostic in colorectal cancer (CRC) and may have a correlation with vascular endothelial growth factor (VEGF) expression via hypoxia-inducible factor 1 (HIF-1) alpha, and the PI3K/mTOR pathways. We therefore have explored the prognostic association of PTEN loss and the potential that PTEN loss may be predictive of outcome with bevacizumab. Patients enrolled in the AGITG MAX trial, a randomized Phase III trial of capecitabine (C) +/− bevacizumab (B) (+/− mitomycin C [M]) with available tissues were analyzed for PTEN expression (loss vs. no loss) as assessed using a Taqman® copy number assay (CNA). Of the original 471 patients enrolled, tissues from 302 (64.1%) patients were analyzed. PTEN loss was observed in 38.7% of patients. There was no relationship between PTEN loss and KRAS or BRAF mutation. PTEN status was not prognostic for progression-free survival (PFS) or overall survival (OS) in multivariate analyses adjusting for other baseline factors; loss versus no loss PFS hazard ratio (HR) 0.9 (0.7–1.16), OS HR 1.04 (0.79–1.38). PTEN was not prognostic when assessed by *KRAS* and *BRAF* status. By using the comparison of C versus CB+CBM, PTEN status was not significantly predictive of the effectiveness of B for PFS or OS. PTEN status was not prognostic for survival in advanced colorectal cancer, irrespective of *KRAS* or *BRAF* status. PTEN status did not significantly predict different benefit with bevacizumb therapy.

## Introduction

Treatment of cancer as a whole has evolved over recent years with the development of so called targeted therapies based on our greater understanding of molecular pathways in cancer. For colorectal cancer (CRC) the major advances have involved drugs that target the epidermal growth factor receptor (EGFR) and vascular endothelial growth factor (VEGF) [1]. As only a subset of patients derives benefit from targeted agents, a major focus of current research is the search for biomarkers which may predict patients who may have greater degrees of sensitivity to a particular targeted agent. For anti-EGFR monoclonal antibodies, the accepted predictors include KRAS [2, 3] and potentially BRAF [4]. There remains, however, an urgent need for biomarkers for anti-VEGF therapies.

The tumor suppressor gene PTEN (phosphatase and tensin homologue deleted on chromosome 10) is an important negative regulator of the PI3K/AKT pathway and controls cell proliferation, survival, and angiogenesis. In patients with metastatic colorectal cancer (mCRC), *PTEN* gene mutation has been reported in 2–20%, with higher rates in microsatellite stable groups [5], while loss of PTEN protein has been reported in 13–55% [6–10]. There is debate as to the best method of reporting low PTEN expression/loss of function, with the most frequent method reported being immunohistochemistry (IHC) [4], although more recently copy number alterations (CNA) in PTEN have been used to assess prognosis in prostate cancer [11]. Furthermore, recent reports have complicated matters further by differentiating roles of cytoplasmic and nuclear PTEN [12]. The prognostic value of PTEN loss in mCRC also remains controversial. Results have thus far been based on small patient numbers, mostly involving patients receiving anti-EGFR antibodies, and have been somewhat inconsistent. Laurent-Puig et al. [13] found that loss of PTEN expression was associated with poorer OS in a KRAS wild-type (WT) population, who had received cetuximab plus or minus irinotecan. A further analysis of primarily Stage II and III CRC also suggested loss of PTEN expression is associated with worse outcome, but primarily in the Stage II group [14]. In contrast, Loupakis et al. [15] failed to confirm an association of PTEN and outcome and, furthermore, showed that PTEN status varies between primary and secondary tumor samples, further complicating interpretation of the data.

PTEN is thought to have a potential role as a biomarker for anti-EGFR therapy in CRC, although the results are not consistent. Relevant to this patient group, there is evidence that decreased levels of PTEN results in increased expression of VEGF, suggesting a potential relationship of outcomes and anti-VEGF therapy [16]. One suggested mechanism is that PTEN loss results in unopposed PI3K activity, which in turn may promote VEGF effects on endothelial cells. This effect is particularly the case in hypoxia where up regulation of hypoxia-inducible factor 1 (HIF-1) alpha by PI3K/mTOR activation results in increased VEGF expression [17, 18].

Given the potential interaction between loss of PTEN expression and VEGF pathway activation and importantly the uncertainty in relation to its impact on prognosis, we undertook an analysis of tumor samples collected during the course of the AGITG MAX trial, which involved patients with advanced colorectal cancer receiving chemotherapy with or without the anti-VEGF antibody bevacizumab. We aimed to evaluate the prognostic impact of PTEN loss based on CNA, as well as determining the potential for predictive outcomes in patients receiving bevacizumab treatment. We also assessed the interaction of PTEN loss and KRAS and BRAF status on prognosis and outcome with bevacizumab.

## Methods and Patients

### Patients and study design

The MAX study design and eligibility criteria have been reported previously [19]. The primary objective of this Phase III study was to evaluate the effect of adding bevacizumab with or without mitomycin C to capecitabine on progression-free survival (PFS) among patients receiving first line chemotherapy for their mCRC. Enrollment occurred between July 2005 and June 2007. Patients were randomly assigned to receive capecitabine (C), capecitabine and bevacizumab (CB), and capecitabine, bevacizumab, and mitomycin C (CBM). Patients were evaluated for tumor response or progression every 6 weeks. Treatment was continued until the disease progressed or until the patient could not tolerate the toxic effects. All patients participating in translational studies provided written informed consent at the time of study enrolment. Ethics approval for translational studies was obtained centrally. To assess whether PTEN was predictive of bevacizumab treatment efficacy, a proportional hazards model with treatment covariate (C vs. CB and CBM), PTEN expression, and their interaction was assessed. To determine whether PTEN was an independent prognostic factor, a multivariate proportional hazards regression model was fitted to data for all patients, with PTEN CNA and other trial protocol pre-specified baseline covariates in the model.

### Tumor collection and processing

Formalin-fixed, paraffin-embedded (FFPE) samples of tumor tissue from archival specimens collected at the time of diagnosis were retrieved from storage at hospital pathology departments. Genomic DNA was isolated from 1 to 2 FFPE tissue sections (10 μm) from each case mounted on a plain glass slide, with an adjacent section stained with haematoxylin and eosin for reference. Cases were reviewed by a histopathologist and if deemed to have <50% malignant crypts in the section, the tissue was manually dissected to ensure a high proportion of tumor cells. Paraffin was removed by xylene and DNA extracted using the QIAamp DNA FFPE tissue kit (Qiagen, Valencia, CA), according to the manufacturer's protocol. Researchers who assessed PTEN copy number were blinded from clinical endpoints. As per the protocol, loss of PTEN is defined as ≤1.5 copies, no loss was >1.5 copies.

### PTEN analyses

All available tissue samples were analyzed for PTEN copy number as assessed using a Taqman® copy number assay (Life Technologies, Carlsbad, CA) to measure copy number variation at the PTEN locus. The assay is a duplex polymerase chain reaction (PCR) for the *PTEN* gene and the reference gene, RNaseP (normalizer), using 10 ng DNA in quadruplicate PCR according to the supplier's protocol and run on the Rotorgene 6000 real time PCR instrument (Qiagen). The results were calculated as a ratio relative to a 2-copy control using the 2^−ΔΔCt^ method (Rotorgene software), and multiplied by 2 to give the copy number. Loss of PTEN was defined as ≤1.5 copies, no loss was >1.5 copies. We tested DNA from colon cancer cell lines to determine the reproducibility of the assay and to select cell lines (LIM2405, LIM1899, HT29) with known PTEN copy number, to use as 1, 2, and 3 copy controls, respectively. The controls were tested in quadruplicate and repeated in three separate PCR assays. The assay was both precise and reproducible – the means for the controls for 1 copy, 2 copies, and 3 copies were 1.08 SEM 0.04, 2.07 SEM 0.03, and 2.96 SEM 0.07, respectively. The coefficient of variation (CV) between eight runs was 2.4%, and intra-assay CV was between 0.12% and 0.99%.

### KRAS and BRAF analysis

The methods used for assessing KRAS and BRAF mutations have been previously described [20].

### Statistical analysis

All randomly assigned patients for whom data on PTEN expression (loss vs. no loss) were available were included in the analysis. PFS, the primary endpoint, was defined as the time from randomization until documented evidence of disease progression according to the Response Evaluation Criteria in Solid Tumors (RECIST) version 1.0, the occurrence of new disease or death from any cause. The secondary endpoints were overall survival (OS), defined as the time from randomization until death from any cause; and response rate (RR), defined according to RECIST version 1.0.

The PFS of patients according to PTEN expression and treatment groups were summarized with the use of Kaplan–Meier curves, and the difference between these groups was compared (C vs. CB and CBM) with the use of the log-rank test. A proportional hazards model with treatment covariate (C vs. CB and CBM), PTEN expression, and their interaction was used to assess whether PTEN was predictive of bevacizumab treatment efficacy. To assess whether PTEN was an independent prognostic factor, a multivariate proportional hazards regression model was fitted to data for all patients, with PTEN expression and other trial protocol pre-specified baseline covariates in the model. The same methods were used for grouping by KRAS and BRAF, based on the tissue population status as previously published [20].

## Results

### Characteristics of the patients

**Figure 1:**
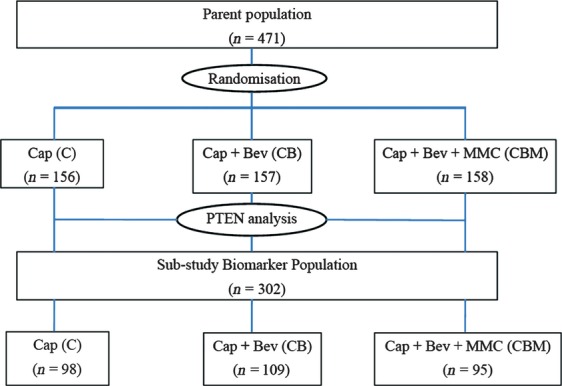
CONSORT diagram. C, capecitabine; CB, capecitabine plus bevacizumab; CBM, CB plus mitomycin.

Of 471 patients who underwent random assignment, a total of 302 tumor specimens were examined for PTEN expression (accounting for 64.1% of the total study population; Fig. 1). Specimen breakdown is as follows: Arm A (C) primary tumors 90, metastasis 4, Lymph node 3, Local recurrence 1 (*n* = 98); Arm B (C+B) primary 100, metastasis 4, Lymph node 3, Local recurrence 2 (*n* = 109); Arm C (C+B+M) primary 92, metastasis 3 (*n* = 95). The median follow-up time of these patients was 30.6 months (range, 0.4–42.4 months). Overall 94% of tissue assessed came from the primary. Tumor specimens from the remaining patients could not be retrieved or were not suitable for analysis. Although a nominal figure of >50% malignant crypts was required in each section as part of the original design, to reduce the impact of normal tissue on the PTEN analysis, minimal normal tissue was allowed and manual dissection ultimately occurred in 70.5% of cases.

Among those patients analyzed for PTEN expression (*n* = 302), PTEN loss was observed in 38.7%. Baseline characteristics by PTEN expression are summarized in Table 1. PTEN loss was more frequent in patients with primary rectal cancer (*P* = 0.01), and patients were less likely to have lung metastasis *(P* = 0.03). There was no association between PTEN loss and KRAS mutation (*P* = 0.24) or BRAF (*P* = 0.89) mutation. Other baseline characteristics were similar between those with and without loss of PTEN expression.

**Table 1:** Baseline patient demographic characteristics

	All patients who underwent randomization (*n* = 471)	%	No PTEN loss (*n* = 185)	%	PTEN loss (*n* = 117)	%	*P*-value
Age (years)							
Median	67		67		69		0.06
Range	32–86		32–85		32–85		
Male	295	63	120	65	68	58	0.24
ECOG performance status							
0	263	56	112	61	66	56	0.44
1	178	38	65	35	42	36	
2	30	6	8	4	9	8	
Capecitabine dosage							
1 g/m^2^ bd	314	67	116	63	83	71	0.14
1.25 g/m^2^ bd	157	33	69	37	34	29	
Disease-free interval > 12 months	125	27	49	27	37	33	0.31
KRAS mutation^1^	90	29	55	29	28	24	0.24
KRAS wild-type^1^	224	71	127	70	89	76	
BRAF mutation^1^	33	11	20	11	12	10	0.89
BRAF wild-type^1^	280	89	163	88	103	88	
Prior adjuvant treatment							
Chemotherapy	104	22	40	22	29	25	0.52
Radiotherapy	59	13	13	7	16	14	0.06
Primary site of cancer							
Caecum	49	10	20	11	8	7	0.25
Ascending colon	47	10	30	16	11	9	0.09
Transverse colon	28	6	13	7	5	4	0.33
Descending colon	16	3	5	3	6	5	0.27
Sigmoid colon	139	30	52	28	39	33	0.34
Recto-sigmoid colon	54	11	26	14	11	9	0.23
Rectum	107	23	29	16	32	27	0.01
Other	27	6	9	5	5	4	0.81
Uncertain	4	1	1	1	0	0	0.43
Primary tumor resected	371	79	161	87	110	94	0.05
Any metastases resected	45	10	14	8	12	10	0.42
Extent of disease at baseline							
Local disease (colon or rectum)	169	36	58	31	29	25	0.22
Liver metastases	353	75	131	71	91	78	0.18
Lymph node metastases	219	47	87	47	53	45	0.77
Lung metastases	185	39	64	35	55	47	0.03
Bone metastases	18	4	6	3	4	3	0.93
Peritoneal metastases	84	18	33	18	17	15	0.45
Other metastases	49	10	24	13	11	9	0.35

ECOG, Eastern Cooperative Oncology Group; PTEN, phosphatase and tensin homologue.

1 Only 66.9% of the total patient population was evaluated for KRAS and BRAF mutations. Not all of these patients were also evaluated for PTEN expression.

Baseline characteristics of those with and without tissues for PTEN analysis were also comparable (Table 1); clinical outcomes were comparable with no significant difference in primary or secondary clinical outcomes between the total study population and the patients who were evaluated for PTEN expression (Tables S1 and S2).

### Progression-free survival

**Figure 2:**
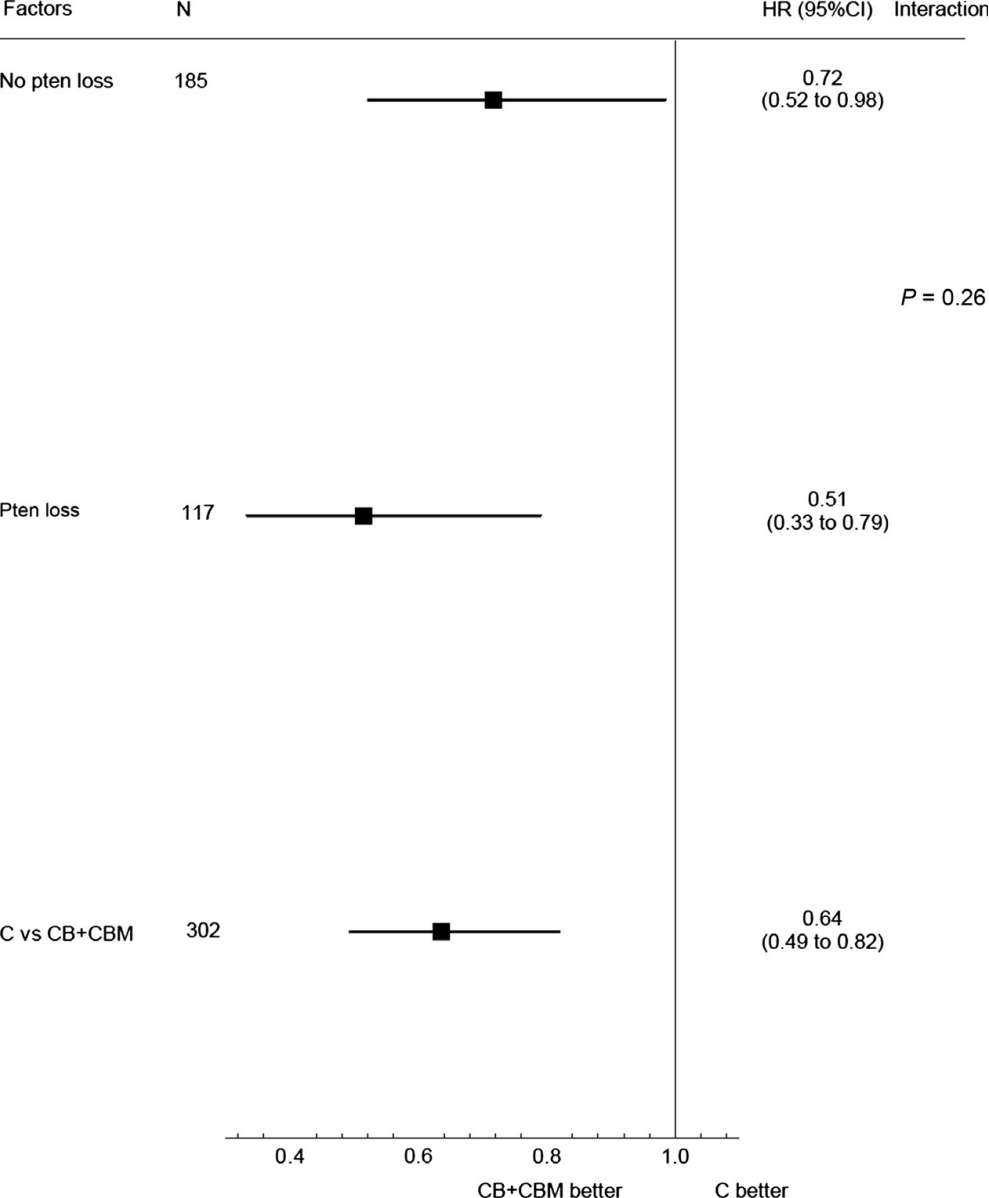
Forest plot to demonstrate hazard ratios (HRs) for progression-free survival subgroup analyses by PTEN status. C, capecitabine; CB, capecitabine plus bevacizumab; CBM, CB plus mitomycin; PTEN, phosphatase and tensin homologue.

Among patients with loss of PTEN expression in tumors, the median PFS was 6.0 months in the group receiving capecitabine and was 9.2 months in the groups receiving CB or CBM. The hazard ratio (HR) of disease progression was 0.51 (95% CI, 0.33–0.79, *P* = 0.002; Fig. 2). Among patients with no PTEN loss in tumors, the median PFS was 6.1 months in the group receiving capecitabine and was 8.4 months in the groups receiving CB or CBM (HR, 0.72; 95% CI, 0.52–0.98; *P* = 0.04; Fig. 2). The additional benefit of bevacizumab on PFS was not significantly greater among the patients with loss of PTEN expression in tumors than among patients with no PTEN loss in tumors (*P* = 0.26 for the interaction between PTEN expression and the assigned treatment).

### Overall survival

Among patients with loss of PTEN expression in tumors, the median OS was 19.1 months in the group receiving capecitabine and was 20.4 months in the groups receiving CB or CBM (HR, 0.75; 95% CI, 0.47–1.19; *P* = 0.22). Among patients with no PTEN loss in tumors, the median OS was 21.4 months in the group receiving capecitabine and was 18.4 months in the groups receiving CB or CBM (HR, 1.00; 95% CI, 0.70–1.43; *P* = 0.99). The effect of the addition of bevacizumab on OS was not significantly greater among the patients with loss of PTEN expression in tumors than among patients with no PTEN loss in tumors (*P* = 0.35 for the interaction between PTEN expression and the assigned treatment).

### Response to treatment

RR that was based on PTEN expression is summarized in Table 2. The effect of the addition of bevacizumab on response was not significantly greater among the patients with loss of PTEN expression in tumors than among patients with no PTEN loss in tumors (*P* = 0.36 for the interaction between PTEN expression and the assigned treatment).

**Table 2:** Response rate by PTEN expression

Treatment	PTEN loss (%)	PTEN no loss (%)	*P*^1^
C	35.5	34.3	0.36
CB	40.5	32.8	
CBM	56.8	39.2	

C, capecitabine; CB, capecitabine and bevacizumab; CBM, capecitabine, bevacizumab, and mitomycin; PTEN, phosphatase and tensin homologue.

1 *P*-value for interaction between biomarker status and the assigned treatment (C vs. CB and CBM).

### Prognostic value of PTEN

**Figure 3:**
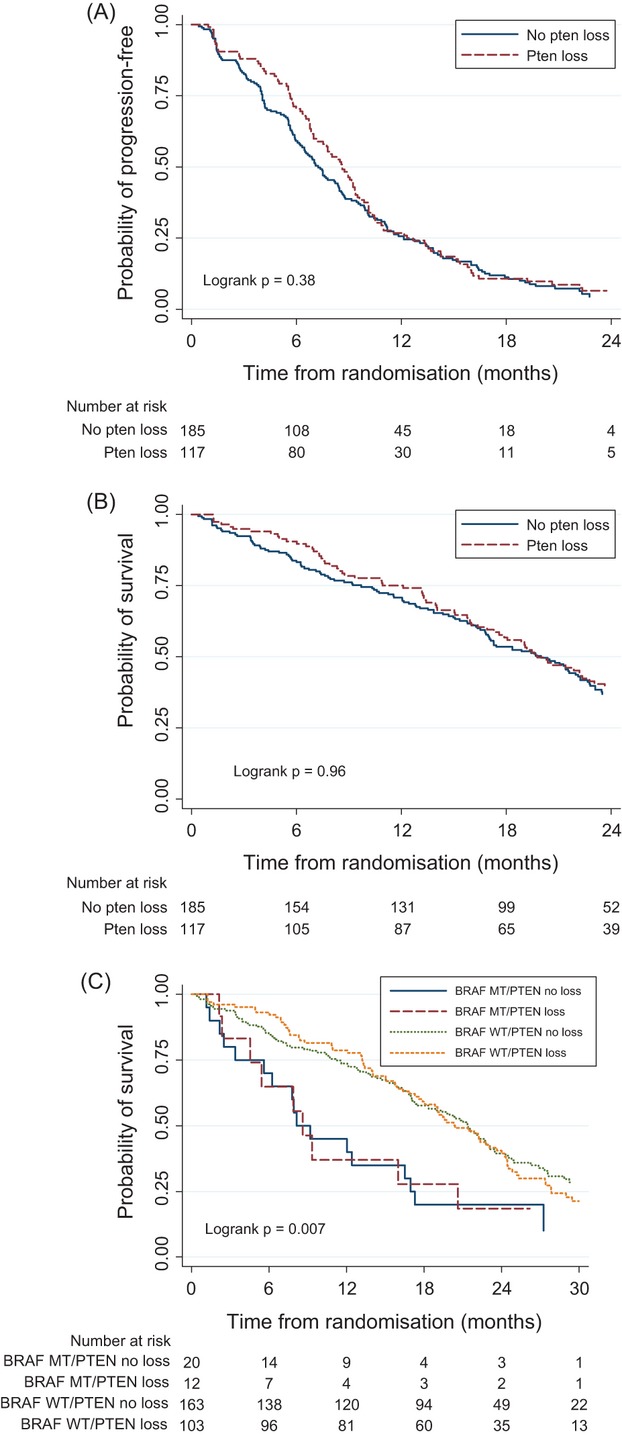
(A) Comparison of progression-free survival of all patients according to PTEN expression; (B) comparison of overall survival of all patients according to PTEN expression; (C) comparison of overall survival of all patients according to PTEN and BRAF expressions. MT, mutated; WT, wild type; PTEN, phosphatase and tensin homologue.

Regardless of treatment arms, PTEN expression did not significantly impact on PFS. The median PFS was 8.6 months among patients with loss of PTEN expression in tumors compared with 7.2 months for patients with no PTEN loss in tumors (HR of PTEN loss vs. no loss, 0.90; 95% CI, 0.70–1.14; *P* = 0.38; Fig. 3A). Similarly, there was no prognostic value noted for PTEN expression on OS (Fig. 3B), with median OS of 19.8 months for those with loss of PTEN expression in tumors compared with 20.0 months for those with no PTEN loss in tumors (HR of PTEN loss vs. no loss, 1.01; 95% CI 0.77–1.32, *P* = 0.96).

In KRAS mutation patients, PTEN expression did not significantly impact on PFS or OS (PFS: median PFS loss vs. no loss, 7.7 vs. 7.4 months; *P* = 0.13; OS: median OS loss vs. no loss, 20.3 vs. 18.4 months; *P* = 0.67). In KRAS wild-type patients, PTEN expression also did not significantly impact on PFS or OS (PFS: median PFS loss vs. no loss, 8.8 vs. 7.2 months; *P* = 0.91; OS: median OS loss vs. no loss, 19.6 vs. 21.1 months; *P* = 0.59).

BRAF mutation is prognostic for poorer OS (median OS mutation vs. wild-type, 8.6 vs. 20.8 months; *P* = 0.001; Fig. 3C). In BRAF mutation patients, PTEN expression did not significantly impact on PFS or OS (PFS: median PFS loss vs. no loss, 7.5 vs. 4.2 months; *P* = 0.53; OS: median OS loss vs. no loss, 8.6 vs. 8.2 months; *P* = 0.91). In BRAF wild-type patients, PTEN expression also did not significantly impact on PFS or OS (PFS: median PFS loss vs. no loss, 8.9 vs. 7.5 months; *P* = 0.40; OS: median OS loss vs. no loss, 20.4 vs. 21.4 months; *P* = 0.66).

Multivariate analyses to adjust for predefined baseline clinico-pathologic prognostic factors did not change these results for the prognostic significance of PTEN expression on both PFS and OS outcomes (results not shown).

## Discussion

Results from the patients with tissue available for PTEN analysis (64.1%) from the randomized Phase III MAX trial show that PTEN status as measured by CNA did not significantly predict differences in benefit from the anti-VEGF agent bevacizumab. PTEN CNA therefore does not appear to be a predictive factor for anti-VEGF therapy in mCRC. Moreover, PTEN CNA was also not found to be prognostic for survival in advanced colorectal cancer.

Loss of PTEN expression (or activity) has been previously assessed as a potential predictive biomarker for resistance to EGFR-targeted monoclonal antibodies in patients with mCRC [4, 21]. IHC is frequently used, although other methods have also been reported (fluorescence in situ hybridization (FISH), mutation status, and CNA) to assess loss of PTEN expression/activity. The accurate determination of PTEN status is difficult, and is not always reliable, particularly when assessed by IHC given the potential for inter reporter variation [22]. Assessment of PTEN expression is further complicated by potential discordance between the expression of PTEN in the primary and metastatic tissue. Concordance rates reported vary from 47% to 89% between primary and secondary tissue [22–25]. FISH may have a role with higher concordance rates of 82% reported, although this study had only 8 patients [8]. Even if concordance rates are improved, reliability is also complicated by the findings of Loupakis et al. [15] who found that PTEN loss in the primary tumor was not predictive of resistance to EGFR-targeted monoclonal antibodies, but it was predictive in metastatic tissue. Newer methods using a Taqman® copy number assay to measure CNA at the PTEN locus in a duplex PCR as used in our study may allow for more reproducible results. There remains a need to compare the various methods assessing PTEN loss, although given the described variations in IHC PTEN assessment it will be difficult to define what is a standardized approach.

In our study, 94% of tissue was from the primary, which may have reduced the impact of tissue source variation and thus allow for more consistent results, although it did not allow us to assess the effect of PTEN loss in metastases on prognosis. Ultimately however, the issues of interpreter variation for IHC and low concordance between primary and metastasis may make routine interpretation of PTEN status difficult in clinical practice and repeat biopsy of new lesions may be required if PTEN is to be used as a robust predictive marker in this setting.

Assessing PTEN status in the setting of anti-VEGF therapy is based on the proposed interaction of the PI3K/PTEN/AKT pathway and VEGF expression [16–18, 26]. Activation of AKT, in part induced by over expression of VEGF itself, leads to angiogenesis [27] and PTEN loss facilitates PI3K expression. HIF-1 (α and β) is also a potent stimulus of VEGF production and although hypoxia itself is a key factor, activation of VEGF potentially via PI3K can also contribute to increased HIF-1α [17]. Anti-VEGF therapy may therefore overcome to a degree these driving factors. Thus one may hypothesize that the addition of bevacizumab may be more effective in patients with low or no expression of PTEN. However, our study has shown that PTEN loss does not appear to affect bevacizumab efficacy based on PFS, RR, or OS. Thus PTEN copy number loss is not a predictive factor for bevacizumab therapy when combined with capecitabine in metastatic CRC.

Prognostically, PTEN loss, with its subsequent downstream effect on the PI3K/AKT pathway, may impact on outcome given the stimulation of tumorigenesis [26], as well as angiogenesis. Prior reports on the effect of PTEN on prognosis have been variable [6, 7]. For example Laurent-Puig et al. [13] primarily report on the KRAS WT group receiving cetuximab rather than the whole population. Although in this specific group they found loss of PTEN associated with poor OS, we also assessed this subgroup and failed to confirm this result in a patient population not receiving an anti-EGFR agent. This suggests that their findings may relate more to the impact of PTEN on predicting outcomes to anti-EGFR agents. Hsu et al. [14] also studied PTEN loss predominantly in early stage disease (Stage II and III) and suggested a prognostic effect but, primarily in the Stage II population. This has also been reported when assessing the relationship of *KRAS* status and prognosis with conflicting results and differences based on stage noted. For example, the RASCAL cooperative was for all stages of colorectal cancer and 12 possible mutations on codons 12 and 13 of *KRAS* were assessed [28]. Only one mutation on codon 12, glycine to valine, found in 8.6% of all patients, had a statistically significant impact on PFS (*P* = 0.004, HR 1.3) and OS (*P* = 0.008, HR 1.29) and the impact on outcome appeared to be greater in Dukes' C cancers (PFS, *P* = 0.008, HR 1.5; OS *P* = 0.02, HR 1.45) rather than in Dukes' B tumors (PFS, *P* = 0.46, HR 1.12; OS *P* = 0.36, HR 1.15) and there was no obvious signal in mCRC. Ultimately our results from a large dataset of patients with metastatic disease do not support loss of PTEN expression based on CNA as a prognostic marker in the overall population or any subgroups based on KRAS or BRAF mutation status.

In conclusion, PTEN CNA cannot be considered a predictive factor for anti-VEGF therapy with bevacizumab, or a prognostic factor for mCRC. There remains a need to further explore potential markers of outcome for anti-VEGF therapy to better select patients best treated with this class of drug and additional studies are being undertaken on the MAX study tissue population with this aim.

## Supporting Information

**Table S1.** Progression-free survival in the total study population and the patients evaluated for PTEN expression.

**Table S2.** Overall survival in the total study population and the patients evaluated for PTEN expression.
