# Supplementary material for: Prognostic impact and the relevance of PTEN copy number alterations in patients with advanced colorectal cancer (CRC) receiving bevacizumab
Source: Cancer Med. 2013 Mar 25;2(3):277–85. doi: 10.1002/cam4.75 (PMC3699839; doi:10.1002/cam4.75)
Supplement: Supplementary file 1 [file cam40002-0277-SD1.docx]

Supplementary Table 1 – Progression-Free Survival in the Total Study Population and the Patients Evaluated for PTEN expression

|  | All patients who underwent randomisation (n=471 ) | | | | Patients evaluated for PTEN expression (n=302) | | | |
| --- | --- | --- | --- | --- | --- | --- | --- | --- |
| Treatment | Median PFS (mths) | HR | 95% CI | p-value | Median  PFS (mths) | HR | 95% CI | p-value |
| C | 5.7 | 1.00 |  |  | 6.0 | 1.00 |  |  |
| CB | 8.5 | 0.62 | 0.50 - 0.79 | <0.0001 | 9.1 | 0.65 | 0.49 - 0.87 | 0.003 |
| CBM | 8.4 | 0.59 | 0.47 - 0.75 | <0.0001 | 8.5 | 0.62 | 0.46 - 0.83 | 0.002 |
|  |  |  |  |  |  |  |  |  |
| C | 5.7 | 1.00 |  |  | 6.0 | 1.00 |  |  |
| CB +CBM | 8.4 | 0.61 | 0.50 - 0.74 | <0.0001 | 8.6 | 0.64 | 0.49 - 0.82 | <0.0001 |

Supplementary Table 2 – Overall Survival in the Total Study Population and the Patients Evaluated for PTEN expression

|  | All patients who underwent randomisation (n=471 ) | | | | Patients evaluated for PTEN expression (n=302) | | | |
| --- | --- | --- | --- | --- | --- | --- | --- | --- |
| Treatment | Median OS (mths) | HR | 95% CI | p-value | Median  OS (mths) | HR | 95% CI | p-value |
| C | 18.9 | 1.00 |  |  | 20.0 | 1.00 |  |  |
| CB | 18.9 | 0.86 | 0.66 – 1.11 | 0.24 | 19.8 | 0.91 | 0.66 - 1.25 | 0.56 |
| CBM | 16.4 | 1.00 | 0.78 – 1.29 | 0.98 | 19.7 | 0.90 | 0.64 - 1.25 | 0.53 |
|  |  |  |  |  |  |  |  |  |
| C | 18.9 | 1.00 |  |  | 20.0 | 1.00 |  |  |
| CB +CBM | 17.3 | 0.93 | 0.75 – 1.16 | 0.51 | 19.8 | 0.90 | 0.68 - 1.20 | 0.49 |
